# Longitudinal assessment of U-shaped and inverted U-shaped developmental changes in the spontaneous movements of infants via markerless video analysis

**DOI:** 10.1038/s41598-020-74006-y

**Published:** 2020-10-08

**Authors:** Naoki Kinoshita, Akira Furui, Zu Soh, Hideaki Hayashi, Taro Shibanoki, Hiroki Mori, Koji Shimatani, Yasuko Funabiki, Toshio Tsuji

**Affiliations:** 1 Graduate School of Engineering, Hiroshima University, 1-4-1 Kagamiyama, Higashi-Hiroshima, Hiroshima, 739-8527 Japan; 2 Graduate School of Advanced Science and Engineering, Hiroshima University, 1-4-1 Kagamiyama, Higashi-Hiroshima, Hiroshima, 739-8527 Japan; 3 Faculty of Information Science and Electrical Engineering, Kyushu University, 744 Motooka, Nishi-Ku, Fukuoka, 819-0395 Japan; 4 College of Engineering, Ibaraki University, 4-12-1 Nakanarusawa, Hitachi, Ibaraki 316-8511 Japan; 5 Future Robotics Organization, Waseda University, 1-104 Totsuka, Shinjuku-Ku, Tokyo, 169-8050 Japan; 6 Department of Physical Therapy, Prefectural University of Hiroshima, 1-1 Gakuen, Mihara, Hiroshima, 723-0053 Japan; 7 Graduate School of Human and Environmental Studies, Kyoto University, Yoshida-Nihonmatsu-Cho, Sakyo-Ku, Kyoto, 606-8507 Japan

**Keywords:** Physical examination, Paediatric research, Paediatric research

## Abstract

Various attempts have been made to elucidate the development patterns in the spontaneous movements of infants through longitudinal evaluations. Movement complexity has been found to demonstrate u-shaped changes in the measurements focusing on limb movements. However, researchers have not yet clarified how other characteristics, besides movement complexity, change over time. This paper presents a longitudinal evaluation of spontaneous movements in infants using evaluation indices calculated through markerless video analysis. Nine infants with corrected ages from $-1$ to 15 weeks participated in the experiments. We confirmed the change in indices over time using statistical methods. Index changes can be classified as positively correlated, u-shaped, inverted u-shaped, and uncorrelated. We also confirmed that the u-shaped and inverted u-shaped indices are negatively correlated. Furthermore, the principal component analysis revealed that the first principal component had the inverted u-shaped changes with the corrected age. These results suggest that it is important to synchronize the inverted u-shaped variations in the movement and velocity with the u-shaped changes in the movement complexity for infant development.

## Introduction

Movement abnormality in infants has been linked to neurological disorders^1,2^. Spontaneous limb movement observations have been reported to be especially useful for neurological evaluations^3–5^. General movements (GMs) are a part of the spontaneous movement repertoire and are used for neurological evaluation. The evaluation of GMs can predict the prognosis of a disorder almost as effectively as a neurological examination^6^. A report stated that 98% of the 44 infants who did not exhibit fidgety movements, a type of normal GMs, were diagnosed with severe cerebral palsy 2 years later^7^. Therefore, observing spontaneous movements during infancy is effective for the developmental evaluation and early detection of disabilities.

To detect the developing problems in infants at the earliest, researchers have attempted to elucidate the developmental patterns of infants via the longitudinal evaluation of spontaneous movements^8–12^. For example, Piek and Gasson discovered strong synchronization in both legs from the longitudinal evaluation of lower-limb movements, suggesting that variability in the leg movements of infants decreases as they develop^10^. By analyzing the limb movement trajectory, Taga et al. found that the movement complexity shows a u-shaped change; moreover, they reported that this u-shaped change was not observed in infants with brain injury^11^. Gima et al. also confirmed a u-shaped change in the lower-limb movement and reported that this phenomenon occurs so that infants can acquire more developed movements^12^. However, there is a lack of research clarifying how characteristics other than the movement complexity change over time or why the u-shaped changes in movement complexity are necessary for movement development.

Meanwhile, attempts have been made in recent years to evaluate spontaneous movements using markerless video analysis, thereby reducing the measurement costs and the burden on the observed infants. Adde et al. calculated evaluation indices, such as the quantity of whole-body movement, from video images and demonstrated the possibility of predicting cerebral palsy^13^. Moreover, Tsuji et al. proposed a markerless video analysis system that can calculate 25 evaluation indices, characterizing limb and whole-body movements, and demonstrated that the types of GMs can be identified with high accuracy using machine learning^14^; however, this system has only been used for cross-sectional evaluation instead of longitudinal evaluation. The evaluation of longitudinal changes in the spontaneous movements accompanying infant development may help in further clarifying the developmental patterns in infants and possibly elucidate the role and meaning of u-shaped changes in spontaneous movement complexity for movement development.

In this study, we analyzed video images taken over a long period and calculated the evaluation indices using the markerless movement measurement and evaluation system proposed by Tsuji et al. This system enables the longitudinal evaluation of spontaneous movements without the need to attach markers to the bodies of infants. The relationship between the changes in each index and the development process was examined through a significance test on the evaluation index values with the corrected ages of infants. In addition, the 25 calculated indices were compressed via principal component analysis (PCA) to interpret their meanings for infant development. This study aimed to evaluate infant developmental patterns both objectively and quantitatively.

**Table 1:** Measurement period and number of measurements.

Participant	Corrected age (weeks)																
	– 1	0	1	2	3	4	5	6	7	8	9	10	11	12	13	14	15
A	–	–	4	5	2	2	2	5	4	5	3	6	3	5	3	4	5
B	–	–	–	4	4	4	4	4	4	4	4	4	4	4	4	4	4
C	–	–	–	–	–	1	4	2	3	6	–	6	3	5	4	5	2
D	5	2	1	2	2	1	3	3	2	5	3	4	–	–	–	–	–
E	–	–	–	–	2	1	3	3	2	4	2	3	3	1	3	3	3
F	–	–	–	–	–	–	2	2	2	2	2	2	2	2	2	2	–
G	–	–	1	1	1	1	1	–	1	1	1	1	1	1	2	1	–
H	–	–	1	1	1	–	–	–	–	1	1	1	1	1	1	1	–
I	–	–	–	–	1	1	1	–	–	–	–	1	1	–	1	–	1

## Methods

**Figure 1:**
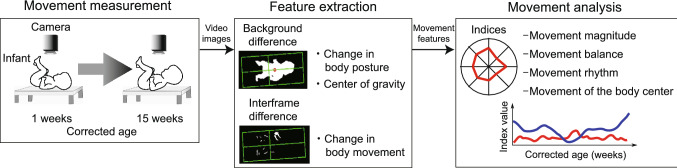
Movement measurement method and analysis procedures. An infants’ movement is recorded without markers for a long time. In feature extraction, image processing is used to capture the characteristics of the recorded movement. In movement analysis, the evaluation indices are calculated using medical knowledge.

We conducted experiments to measure the longitudinal changes observed in the spontaneous movements in infants. Figure 1 shows the procedures followed in these experiments.

### Participnats

Nine infants (Participants A–I) participated in this study and markerless measurements were taken of their spontaneous movements from $-1$ to 15 weeks of their corrected age. All participants were full-term infants (born between a gestation period of 37 and 41 weeks). The birth weights of all participants ranged between 2500 and 4000 g, and there were no complications during the perinatal period. The experiments were conducted in accordance with the Declaration of Helsinki and were approved by the Ethics Committee of Hiroshima University (Registration Number: E-1150-1). Informed consent was obtained from the parents of each infant, and the experimental purpose and methods were fully explained to them. The experiments were also conducted in accordance with the relevant guidelines and regulations. We took appropriate precautions to prevent any risk or burden on the infants.

### Video-based movement analysis

#### Movement measurement

We recorded the infants in a supine position without any marker using a single video camera (HDR-PJ760V, Sony, Japan) situated just above them. The video camera was fixed directly above and parallel to the crib surface, which was covered by a unicolor fabric spread. The video images were stored on a personal computer with a frame rate of 30 Hz. The infants were recorded approximately twice a week until 15 weeks of corrected age (see Table 1). In our video image analysis, we used the periods during which the infants could be seen to be moving (mean: $249.7\pm 145.5$ s; median: 180 s; range: 30–690 s), avoiding any period of sleeping and crying. We also excluded the periods that seemed to have external stimuli, such as parental interference, from the analysis.

#### Feature extraction

First, a background difference image was created from the measurement image. Using this image, we calculated the changes in the body posture $^{(A_{k})}P_l$, expressing the size of the infants’ body as follows:

1 $$\begin{aligned} ^{(A_{k})}P_{l}& = {} {\sum _{(x,\; y) \in {A_{k}}}}{{O_{l}(x,\; y)}}, \end{aligned}$$

where $A_{k}\ (k=1,\; 2,\; \ldots ,\; 9)$ represents the area of the infants’ body. The left upper limb, right upper limb, left lower limb, and right lower limb are denoted as ${A_1}$, ${A_2}$, ${A_3}$, and ${A_4}$, respectively. By combining these four areas, the upper and lower body, the left and right sides, and the whole body were determined as ${A_5}$, ${A_6}$, ${A_7}$, ${A_8}$, and ${A_9}$, respectively. In addition, $(x,\; y)\ (x = 1,\; 2,\; \ldots ,\; W;\; y = 1,\; 2,\; \ldots ,\; H)$ are the image coordinates, $l\ (l = 1,\; 2,\; \ldots ,\; L)$ is the frame number of the image in the video, and ${O_{l}(x,\; y)}$ is the pixel value of the background difference image (where $O_{l}(x,\; y)=1$, if the pixel is white, and $O_{l}(x,\; y)=0$, if it is black). Moreover, using the image center of gravity (COG) calculated from the background difference image, the COG velocity ($G_{l,\; \mathrm{x}} ^{\mathrm{{v}}}$, $G_{l,\; \mathrm{y}}^{\mathrm{{v}}}$), which is the distance moved by the COG between adjacent frames, and COG fluctuations, which is the change in COG from the average COG position ($G_{l,\; \mathrm{x}}^{\mathrm{{d}}}$, $G_{l,\; \mathrm{y}}^{\mathrm{{d}}}$) between $1\le l\le L$, were calculated using the following equations:

2 $$\begin{aligned} \left( G^{\mathrm{{v}}}_{l,\mathrm {x}},\; G^{\mathrm{{v}}}_{l,\mathrm {y}}\right)& = {} \left( \frac{G_{l, \mathrm {x}} - G_{{l-1}, \mathrm {x}}}{\root \of {^{(A_9)}P^{\mathrm{{avg}}}}} , \frac{G_{l, \mathrm {y}} - G_{{l-1}, \mathrm {y}}}{\root \of {^{(A_9)}P^{\mathrm{{avg}}}}}\right) , \end{aligned}$$

3 $$\begin{aligned} \left( G^{\mathrm{{d}}}_{l,\mathrm {x}},\; G^{\mathrm{{d}}}_{l,\mathrm {y}}\right)& = {} \left( \frac{G_{l, \mathrm {x}} - G^{\mathrm{{avg}}}_{\mathrm{{x}}}}{\root \of {^{(A_9)}P^{\mathrm{{avg}}}}} , \frac{G_{l, \mathrm {y}} - G^{\mathrm{{avg}}}_{\mathrm{{y}}}}{\root \of {^{(A_9)}P^{\mathrm{{avg}}}}}\right) , \end{aligned}$$

where $^{(A_9)}P^{\mathrm{{avg}}}$ is an average value from the maximum value to the *E*-th value ($E \le L$) in $^{(A_{9})}P_{l}$ between $1\le l\le L$. It should be noted that $G^{\mathrm{{v}}}_{1,\mathrm {x}}=0$ and $G^{\mathrm{{v}}}_{1,\mathrm {y}}=0$. $G_{l, \mathrm {x}}$ and $G_{l, \mathrm {x}}$ represents the COG coordinates calculated from the background difference image by the following equation:

4 $$\begin{aligned} G_{l, \mathrm {x}}& = {} \frac{1}{^{(A_9)}P_{l}}\sum ^{W}_{x=1}\sum ^{H}_{y=1}{x{O_{l}(x,\; y)}}, \end{aligned}$$

5 $$\begin{aligned} G_{l, \mathrm {y}}& = {} \frac{1}{^{(A_9)}P_{l}}\sum ^{W}_{x=1}\sum ^{H}_{y=1}{y{O_{l}(x,\; y)}}. \end{aligned}$$

Next, an interframe difference image was created from the measurement image and motor alteration $^{(A_{k})}M_l$, expressing the magnitude of the movement with respect to the body size; it can be calculated as follows:

6 $$\begin{aligned} ^{(A_{k})}M_{l}& = {} \frac{\displaystyle {\sum _{(x,\; y) \in {A_{k}}}}{O'_{l}(x,\; y)}}{^{(A_9)}P^{\mathrm{{avg}}}},\nonumber \\ O'_{l}(x,\; y)& = {} |{O_{l}(x,\; y)}-{O_{l-1}(x,\; y)}|, \end{aligned}$$

where $$O'_{1}(x,\; y)=0$$.

The analysis parameters were set to $L=900$ and $E=10$. We used the system developed by Tsuji et al. for feature extraction and movement analysis^14^. Because *W* and *H* depend on the measurement environment, they differ for each image.

#### Movement analysis

By using medical knowledge and the features of the extracted movements, evaluation indices, such as the movement magnitude, balance, and rhythm, were calculated. Details for calculating each evaluation index are provided in Supplementary Material. In this analysis, we used some of the indices proposed by Tsuji et al. (see Table 2). In the table, ${A_5}$, ${A_6}$, and ${A_9}$ represent the upper-, lower-, and whole-body areas of an infant, respectively. The evaluation indices were calculated every 30 s; subsequently, the average daily values were calculated. In addition, each index was standardized by calculating its average value and standard deviation within each participant.

### Analysis of longitudinal changes in movements

The changes in each evaluation index with the corrected age were statistically analyzed. The developmental changes have been reported as linear and u-shaped^11^; therefore, linearity and u-shaped tests were performed on the evaluation indices with the corrected age. In the linearity test, the significance of the correlation coefficient between an evaluation index and corrected age was tested with a significance level of 5%. In the u-shaped test, a two-lines test^15^ was used, wherein an extreme value of the u-shaped change was first calculated based on the approximation by a quadratic function. The significance of the correlation coefficient was then tested for each side (right and left) of the data, separated by the extreme value. A significant u-shaped change was observed if the two correlation coefficients had opposite signs and were individually significant. When the two-lines test demonstrated a change from a decrease to an increase, with the extreme value as the boundary, the trend was defined as u-shaped, and when it showed a change from an increase to a decrease, it was defined as inverted u-shaped. If significant results were obtained for both the linearity and u-shaped tests, the trend that yielded smaller residual sum of squares among the approximate straight lines was adopted. If the results were not significant, the index was classified to be uncorrelated with the corrected age.

PCA was performed to reduce and interpret the information obtained from the 25 indices. Let ${\mathbf {X}} \in {\mathbb {R}}^{n \times 25}$ ($n=272$: 272 is the number of samples) be the matrix in which the 25 indices for all participants are arranged chronologically. Note that each index in ${\mathbf {X}}$ was standardized over all participants to have a mean of zero and unit variance. PCA was then conducted via singular value decomposition using the following equation:

7 $$\begin{aligned} {\mathbf {X}}={\mathbf {U}} \mathbf{\Sigma } {\mathbf {W}}^{\mathrm{T}}, \end{aligned}$$

where ${\mathbf {U}} \in {\mathbb {R}}^{n \times n}$ and ${\mathbf {W}} \in {\mathbb {R}}^{25 \times 25}$ are square matrices and $\Sigma \in {\mathbb {R}}^{n \times 25}$ is a rectangular diagonal matrix. The columns of ${\mathbf {W}}$ denote the eigenvectors of the covariance matrix and represent the loading vector of the principal component space. ${\mathbf {U}} \mathbf{\Sigma }$ is the principal component score and represents the projection of ${\mathbf {X}}$ on the principal component space. PCA was used to examine the relationship between the principal component scores with the highest contribution rate and corrected age. We also evaluated the principal component space by confirming the indices with large loadings. The principal components extracted through PCA were selected based on the Kaiser criteria^16^, with an eigenvalue greater than one.

**Table 2:** Description of evaluation indices^14^.

Category	Index	Description
Movement magnitude	${^{(A_5)}I_1}$, ${^{(A_6)}I_1}$	Movement frequency
	${^{(A_5)}I_2}$, ${^{(A_6)}I_2}$	Movement strength
	${^{(A_5)}I_3}$, ${^{(A_6)}I_3}$	Movement count
Movement balance	${^{(A_5,\; A_6)}I_4}$	Ratio of index ${^{(A_5)}I_1}$ in the upper body and index ${^{(A_6)}I_1}$ in the lower body
	${^{(A_5,\; A_6)}I_5}$	Ratio of index ${^{(A_5)}I_2}$ in the upper body and index ${^{(A_6)}I_2}$ in the lower body
	${^{(A_5,\; A_6)}I_6}$	Symmetry in the upper and lower body
Movement rhythm	${^{(A_5)}I_7}$, ${^{(A_6)}I_7}$	Rhythm of motor alteration *M*
	${^{(A_5)}I_8}$, ${^{(A_6)}I_8}$	Standard deviations of indices ${^{(A_5)}I_{7}}$ and ${^{(A_6)}I_7}$
	$^{(A_9)}I_{9_{\mathrm{x}}}$, ${^{(A_9)}I_{9_{\mathrm{y}}}}$	Rhythm of the COG velocity
	$^{(A_9)}I_{10_{\mathrm{x}}}$, $^{(A_9)}I_{10_{\mathrm{y}}}$	Standard deviations of indices $^{(A_9)}I_{9_{\mathrm{x}}}$ and ${^{(A_9)}I_{9_{\mathrm{y}}}}$
	${^{(A_9)}I_{11_{\mathrm{x}}}}$, $^{(A_9)}I_{11_{\mathrm{y}}}$	Rhythm of the COG fluctuations
	$^{(A_9)}I_{12_{\mathrm{x}}}$, $^{(A_9)}I_{12_{\mathrm{y}}}$	Standard deviations of indices ${^{(A_9)}I_{11_{\mathrm{x}}}}$ and $^{(A_9)}I_{11_{\mathrm{y}}}$
COG movement	$^{(A_9)}I_{13_{\mathrm{x}}}$, $^{(A_9)}I_{13_{\mathrm{y}}}$	Variations in the COG velocity
	$^{(A_9)}I_{14_{\mathrm{x}}}$, $^{(A_9)}I_{14_{\mathrm{y}}}$	Variations in the COG fluctuations

## Results

**Figure 2:**
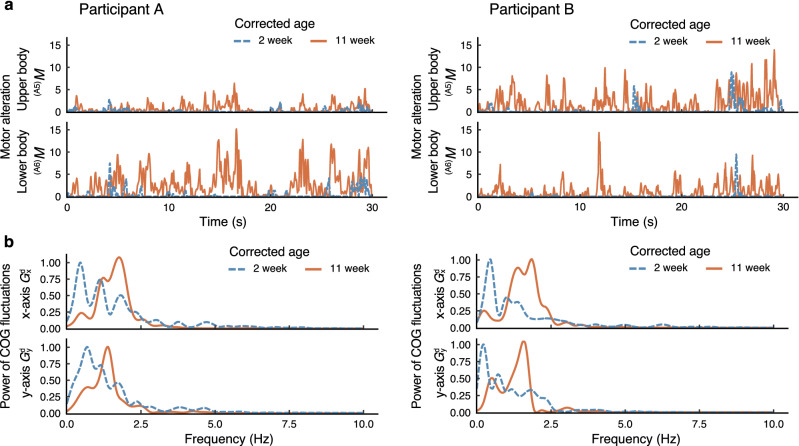
Examples of the features of Participants A and B. (**a**) Motor alteration. (**b**) Power spectral density of COG fluctuations. Each density is normalized so that the maximum value becomes 1. The blue dashed and orange solid lines represent the features at 2 and 11 weeks, respectively.

Figure 2 illustrates examples of the motor alteration (upper body: $^{(A_5)}M$, lower body: $^{(A_6)}M$) and power spectral density of the COG fluctuations (*x*-axis: $G^{\mathrm{d}}_{\mathrm{x}}$, *y*-axis: $G^{\mathrm{d}}_{\mathrm{y}}$) of Participants A and B at 2 and 11 weeks, respectively. Examples for other participants (C–I) are presented in Figs. S1 and S2.

**Figure 3:**
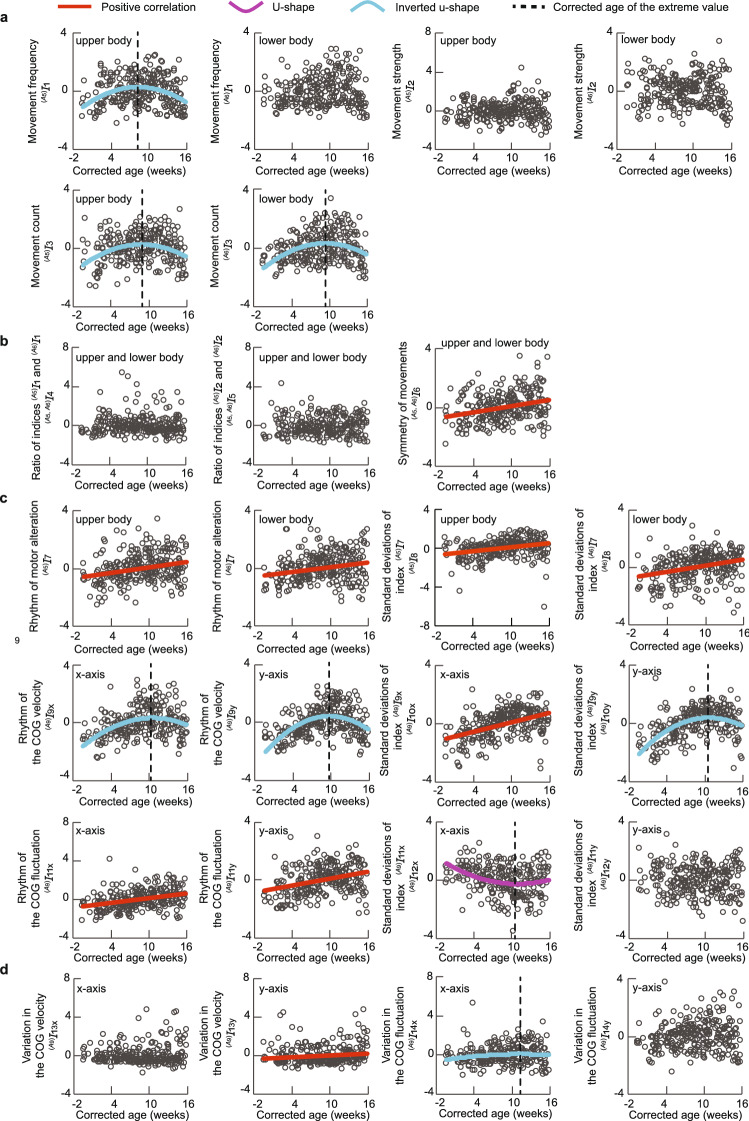
Relationships between the corrected age and each evaluation index in all participants. (**a**) Movement magnitude. (**b**) Movement balance. (**c**) Movement rhythm. (**d**) COG movement. The red, purple, and light blue lines show the changes in the index with positive, u-shaped, and inverted u-shaped correlations to the corrected age, respectively.

Figure 3 shows the changes in each index value with the corrected age in all participants. For indices that produced significant results in the linearity and u-shaped tests, one and two straight lines have been shown in the figure, respectively. Among the 25 indices, 9 positive, 1 u-shaped, and 7 inverted u-shaped correlations were obtained. Eight of the indices were uncorrelated with the corrected age. The mean and standard deviation of the extreme values of the u- and inverted u-shaped changes were 9.92 ± 0.98 weeks, which converged to the same approximate value. Table 3 presents the correlation coefficients and *p* values of the indices for which significant relationships were obtained.

**Table 3:** Correlation coefficient and *p* value in Fig. 3. The u- and inverted u-shaped indices have two correlation coefficients and *p* values, which were calculated for the left- and right-sided data, separated by the extreme value.

Category	Index	Relationship	Correlation coefficient	*p* value
Movement magnitude	${^{(A_5)}I_1}$	Inverted u-shaped	0.230, − 0.295	$1.10\times 10^{-2}$, $2.43\times 10^{-4}$
	${^{(A_6)}I_1}$	Uncorrelated	–	–
	${^{(A_5)}I_2}$	Uncorrelated	–	–
	${^{(A_6)}I_2}$	Uncorrelated	–	–
	${^{(A_5)}I_{3}}$	Inverted u-shaped	0.236, − 0.329	$6.21\times 10^{-3}$, $7.51\times 10^{-5}$
	${^{(A_6)}I_{3}}$	Inverted u-shaped	0.337, − 0.335	$4.10\times 10^{-5}$, $9.72\times 10^{-5}$
Movement balance	${^{(A_5,\; A_6)}I_4}$	Uncorrelated	–	–
	${^{(A_5,\; A_6)}I_5}$	Uncorrelated	–	–
	${^{(A_5,\; A_6)}I_6}$	Positively correlated	0.286	$1.65\times 10^{-6}$
Movement rhythm	${^{(A_5)}I_{7}}$	Positively correlated	0.271	$5.99\times 10^{-6}$
	${^{(A_6)}I_{7}}$	Positively correlated	0.229	$1.39\times 10^{-4}$
	${^{(A_5)}I_{8}}$	Positively correlated	0.287	$1.45\times 10^{-6}$
	${^{(A_6)}I_{8}}$	Positively correlated	0.289	$1.23\times 10^{-6}$
	$^{(A_9)}I_{9_{\mathrm{x}}}$	Inverted u-shaped	0.489, − 0.304	$9.69\times 10^{-12}$, $2.14\times 10^{-3}$
	${^{(A_9)}I_{9_{\mathrm{y}}}}$	Inverted u-shaped	0.608, − 0.438	$1.65\times 10^{-16}$, $4.57\times 10^{-7}$
	$^{(A_9)}I_{10_{\mathrm{x}}}$	Positively correlated	0.457	$2.65\times 10^{-15}$
	$^{(A_9)}I_{10_{\mathrm{y}}}$	Inverted u-shaped	0.563, − 0.410	$4.33\times 10^{-16}$, $3.28\times 10^{-5}$
	${^{(A_9)}I_{11_{\mathrm{x}}}}$	Positively correlated	0.354	$1.86\times 10^{-9}$
	$^{(A_9)}I_{11_{\mathrm{y}}}$	Positively correlated	0.335	$1.49\times 10^{-8}$
	$^{(A_9)}I_{12_{\mathrm{x}}}$	U-shaped	− 0.344, 0.278	$2.58\times 10^{-6}$, $6.63\times 10^{-3}$
	$^{(A_9)}I_{12_{\mathrm{y}}}$	Uncorrelated	–	–
COG movement	$^{(A_9)}I_{13_{\mathrm{x}}}$	Uncorrelated	–	–
	$^{(A_9)}I_{13_{\mathrm{y}}}$	Positively correlated	0.137	$2.40\times 10^{-2}$
	$^{(A_9)}I_{14_{\mathrm{x}}}$	Inverted u-shaped	0.149, − 0.255	$4.03\times 10^{-2}$, $2.17\times 10^{-2}$
	$^{(A_9)}I_{14_{\mathrm{y}}}$	Uncorrelated	–	–

**Figure 4:**
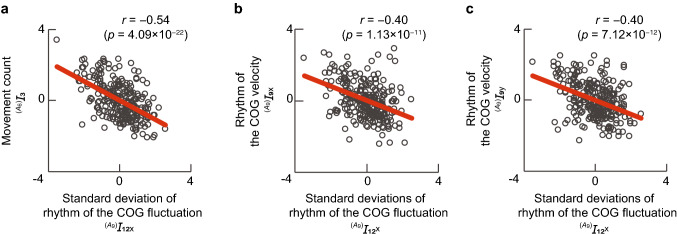
Relationships between the u-shaped and inverted u-shaped indices. (**a**) Movement count (lower limb). (**b**) Rhythm of the COG velocity (x-axis). (**c**) Rhythm of the COG velocity (y-axis). The correlation coefficient and its *p* value are also shown in each figure.

Figure 4 shows the interrelationship between the u-shaped and inverted u-shaped indices. The horizontal axis shows the standard deviations of the rhythm of the COG fluctuations $^{(A_9)}I_{12_{\mathrm{x}}}$, which is the only u-shaped index, and the vertical axis represents the evaluation indices with an inverted u-shaped. Only the three highest correlation coefficient values ($|r|\ge 0.4$) are shown.

**Figure 5:**
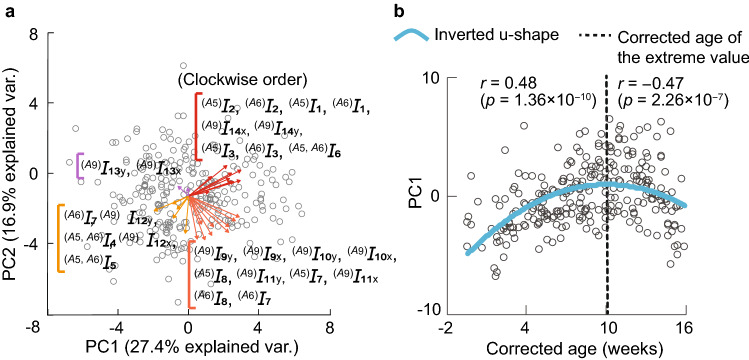
PCA results. (**a**) Biplot of the principal components (PC1 and PC2). Arrows in the figure represent the principal component loadings and the sign of each index. (**b**) Relationships between the corrected age and PC1. The correlation coefficient and its *p* value are also shown for each data side (left and right), separated by the extreme value.

Table 4 presents the principal component loading of each index in the principal component, whose eigenvalue is greater than one according to the Kaiser criteria. Figure 5a illustrates a biplot visualizing the influence of each index on the principal component with respect to the first and second principal components. Figure 5b shows the first principal component score with the corrected age. The correlation coefficient and *p* value obtained after the test are also shown in Fig. 5b.

**Table 4:** PCA results. Eight principal components were selected according to the Kaiser criteria. Values in bold indicate loadings with an absolute value greater than 0.5.

Category	Index	PCA loadings							
		PC1	PC2	PC3	PC4	PC5	PC6	PC7	PC8
Movement magnitude	${^{(A_5)}I_1}$	**0.69**	0.37	− 0.45	0.13	− 0.15	0.02	− 0.15	0.03
	${^{(A_6)}I_1}$	**0.84**	0.42	0.14	− 0.02	0.01	− 0.04	− 0.01	− 0.07
	${^{(A_5)}I_2}$	0.36	0.31	**− 0.71**	-0.01	0.07	− 0.21	− 0.07	− 0.10
	${^{(A_6)}I_2}$	**0.61**	0.49	0.04	− 0.10	0.17	− 0.16	− 0.33	− 0.05
	${^{(A_5)}I_{3}}$	**0.72**	0.23	− 0.20	0.24	− 0.14	0.19	− 0.27	0.07
	${^{(A_6)}I_{3}}$	**0.83**	0.25	0.14	0.07	− 0.02	0.05	0.09	− 0.13
Movement balance	${^{(A_5,\; A_6)}I_4}$	− 0.19	− 0.21	**− 0.66**	0.28	− 0.25	0.17	− 0.16	0.24
	${^{(A_5,\; A_6)}I_5}$	− 0.26	− 0.12	**− 0.80**	0.17	− 0.14	0.02	0.19	− 0.09
	${^{(A_5,\; A_6)}I_6}$	0.32	0.07	− 0.25	− 0.04	0.44	− 0.22	− 0.41	− 0.24
Movement rhythm	${^{(A_5)}I_{7}}$	0.20	− 0.45	0.43	0.42	0.19	0.31	− 0.22	− 0.15
	${^{(A_6)}I_{7}}$	− 0.06	**− 0.60**	− 0.13	0.49	− 0.05	0.34	− 0.09	− 0.08
	${^{(A_5)}I_{8}}$	0.39	− 0.49	0.21	0.39	0.26	− 0.01	− 0.16	− 0.19
	${^{(A_6)}I_{8}}$	0.13	**− 0.69**	− 0.14	0.31	0.12	− 0.12	0.14	− 0.03
	$^{(A_9)}I_{9_{\mathrm{x}}}$	**0.75**	− 0.40	0.01	0.01	0.04	− 0.19	0.12	0.26
	${^{(A_9)}I_{9_{\mathrm{y}}}}$	**0.81**	− 0.34	0.05	− 0.01	− 0.16	− 0.11	0.12	− 0.05
	$^{(A_9)}I_{10_{\mathrm{x}}}$	**0.64**	** 0.58**	− 0.03	− 0.13	0.07	− 0.13	0.04	0.34
	$^{(A_9)}I_{10_{\mathrm{y}}}$	**0.64**	− 0.49	− 0.12	− 0.19	− 0.18	− 0.09	0.15	− 0.26
	${^{(A_9)}I_{11_{\mathrm{x}}}}$	0.26	**− 0.70**	− 0.06	− 0.32	0.22	− 0.14	− 0.14	0.41
	$^{(A_9)}I_{11_{\mathrm{y}}}$	0.37	**− 0.63**	− 0.15	− 0.49	− 0.04	0.10	0.04	− 0.31
	$^{(A_9)}I_{12_{\mathrm{x}}}$	**− 0.56**	− 0.24	− 0.04	− 0.33	0.12	0.14	**− 0.50**	0.24
	$^{(A_9)}I_{12_{\mathrm{y}}}$	− 0.25	− 0.36	− 0.22	**− 0.60**	− 0.05	0.33	− 0.11	− 0.34
COG movement	$^{(A_9)}I_{13_{\mathrm{x}}}$	− 0.02	0.13	− 0.25	− 0.06	**0.70**	0.16	0.22	− 0.09
	$^{(A_9)}I_{13_{\mathrm{y}}}$	− 0.18	0.16	− 0.26	0.10	**0.68**	0.12	0.29	0.12
	$^{(A_9)}I_{14_{\mathrm{x}}}$	**0.65**	0.28	0.06	− 0.18	0.02	0.48	0.08	0.20
	$^{(A_9)}I_{14_{\mathrm{y}}}$	**0.69**	0.23	− 0.03	− 0.18	0.01	**0.50**	0.12	0.15
Proportion of variance explained		0.27	0.17	0.10	0.07	0.06	0.05	0.04	0.04
Cumulative proportion		0.27	0.44	0.54	0.61	0.67	0.72	0.76	0.80

## Discussion

In Fig. 2a, the motor alteration $^{(A_5)}M$, $^{(A_6)}M$ of the upper and lower body at a corrected age of 2 weeks is smaller than that at 11 weeks; it should be noted that the motor alteration of the lower body is very small. Moreover, in Fig. 2b, the power spectral density of the COG fluctuations $(G^{\mathrm{d}}_{\mathrm{x}},\,G^{\mathrm{d}}_{\mathrm{y}})$ at 2 weeks of corrected age was concentrated in the narrow band of the low-frequency range, in comparison to 11 weeks of corrected age. These tendencies were also observed in other participants (see Figs. S1 and S2), and they suggest that as the development progresses, whole-body movements may increase and become more complex by incorporating a variety of velocities.

In Fig. 3, we can observe the changes in the movements of participants with the corrected age; accordingly, the different changes in each index can be confirmed.

An index that demonstrates a positive correlation with the corrected age denotes the symmetry of movements ${^{(A_5,\; A_6)}I_6}$, representing the correlation between the upper- and lower-body movements. This positive correlation indicates that as development progresses, the upper- and lower-body movements become synchronized. We believe that increased movement symmetry can be attributed to lifting legs^17^, which is a spontaneous movement. The lifting of legs increases movements in the lower body as well as the contact between knees and hands. In addition, the rhythm of motor alteration ${^{(A_5)}I_{7}}$, ${^{(A_6)}I_{7}}$ and the rhythm of the COG fluctuation ${^{(A_9)}I_{11_{\mathrm{x}}}}$, ${^{(A_9)}I_{11_{\mathrm{y}}}}$ increased significantly, showing that the movement velocity increases with development. These indices increase owing to spontaneous swiping movements^17^, which have a large amplitude and fast velocity. Moreover, the standard deviations of movement rhythm (${^{(A_5)}I_{8}}$, ${^{(A_6)}I_{8}}$, $^{(A_9)}I_{10_{\mathrm{x}}}$) may increase owing to the increase in fast motions.

It should be noted that only the standard deviations of the rhythm of the COG fluctuations $^{(A_9)}I_{12_{\mathrm{x}}}$ exhibited a u-shaped change, a finding that corroborates previous reports^11^. The changes in this index demonstrate that complicated movements are first simplified, and then become complex again. Therefore, it is possible that the $^{(A_9)}I_{12_{\mathrm{x}}}$ index reflects the conventionally reported u-shaped change.

Seven indices were confirmed to show inverted u-shaped changes. However, the phenomenon of movement change characteristics taking an inverted u-shape with development has not been reported in the past. Therefore, we decided to find the relationship between such indices and conventionally reported the u-shaped changes (see Fig. 4). In Fig. 4, we confirmed that the indices showing u- and inverted u-shaped changes were significantly negatively correlated. The vertical axes of Fig. 4a–c show the movement count ${^{(A_6)}I_{3}}$ and rhythm of the COG velocity $^{(A_9)}I_{9_{\mathrm{x}}}$, $^{(A_9)}I_{9_{\mathrm{y}}}$, which reflect the movement degree and velocity. Therefore, when the movement is complex, it is possible to confirm that a relatively smaller number of slow movements are performed, and when the movement is simple, a larger number of fast movements are performed. Infants must practice and organize many movements to gain mature dexterity; this can be implemented by sending sensations from the body muscles and joints to the brain and integrating them^18^. Hence, infants may increase the sensory input to the brain by performing faster movements when their movements are simpler (i.e., the peak of the u-shaped changes), thereby organizing their movements.

For indices showing no correlation, we confirmed the changes in the indices for each participant. The variations in the COG velocity $^{(A_9)}I_{13_{\mathrm{x}}}$ exhibited a common tendency toward having no correlation with the corrected age in almost all participants; this index may reflect features that do not change with time. By contrast, alternating trends of increase and decrease in the movement strength ${^{(A_5)}I_2}$, ${^{(A_6)}I_2}$ and the ratio of movement strength ${^{(A_5,\;A_6)}I_5}$, which were uncorrelated to the corrected age in all participants, were observed. These indices potentially reflect the large individual differences accompanying the development. In future work, we plan to increase the number of participants and study them in detail.

Figure 5b shows that the first principal component (PC1) with the corrected age acquired an inverted u-shape after being plotted. Figure 5a and Table 4 illustrate that the indices with the largest main component loadings of PC1 included movement frequency ${^{(A_5)}I_1}$, movement count ${^{(A_5)}I_3}$, ${^{(A_6)}I_3}$, and rhythm of COG velocity $^{(A_9)}I_{9_{\mathrm{x}}}$, $^{(A_9)}I_{9_{\mathrm{y}}}$, which all demonstrated an inverted u-shape. Therefore, increasing or decreasing the frequency and velocity of movement to an inverted u-shape at approximately the same time may be important in infant development.

Interestingly, in Fig. 5b, the extreme value of the inverted u-shape occurred at approximately 10 weeks. In healthy infants, 9–10 weeks of corrected age is the time at which writhing GMs transition to fidgety GMs^19^. In general, writhing GMs are movements of small to moderate amplitude and slow to moderate speed, whereas fidgety GMs are movements of smaller amplitude and moderate speed with variable acceleration of the neck, trunk, and limbs^20,21^. The transition from writhing GMs to fidgety GMs occurs at 6–9 weeks of the corrected age, with sporadic fidgety patterns in this period^22–24^. Accordingly, infants gradually acquire faster and more complex movements with the transition from writhing GMs to fidgety GMs. It has also been reported that fidgety GMs become slower and smoother movements at 11–16 weeks of the corrected age after the transition to fidgety GMs^24^. These previous findings correspond with our results stating that the indices related to the frequency and speed of motion that strongly contributed to PC1 increased toward the extreme value and then decreased, i.e., the inverted u-shaped change was observed around the extreme value of 10 weeks. Therefore, the extreme value of the inverted u-shaped PC1 and GM development state may be related. In the future, if we could confirm the loss of inverted u-shape or deviations in their extreme values by analyzing participants who may have an abnormality, we may be able to determine whether the findings obtained in this study can be applied for the early detection of a disorder.

In this study, we analyzed nine infants to confirm the changes in their spontaneous movements from markerless video images. In these experiments, we confirmed that the 25 evaluation indices calculated by the system change with the corrected age. The trends in these changes suggest the possibility of classification as previously reported u-shaped changes, as well as newly reported u-shaped and inverted u-shaped changes. Furthermore, PCA suggested that the major changes over time demonstrate an inverted u-shape.

The length of the video images used in the analysis varied (from 30 to 690 s) depending on the participants and recording date. Relatively long periods were extracted from some of the videos, but in most cases, the data length was 180 s. This is because we excluded the periods when the infants were sleeping and crying, or when external interference was observed, from the analysis. Therefore, although the mood of infants was not completely controlled, we believe that the intrinsic spontaneous movement characteristics of infants could be analyzed with a high degree of sensitivity. To conduct more extensive and detailed analyses in the future, it is necessary to establish an experimental protocol to control the behavioral states efficiently, including the moods of infants, as well as the criteria for selecting videos for the analysis. We also plan to perform measurements at neonatal intensive care units and clarify these developmental patterns in greater detail.

## Supplementary information

Supplementary Information.

## Data Availability

The datasets generated during and/or analysed during the current study are available from the corresponding author on reasonable request.
