# Supplementary material for: Longitudinal assessment of U-shaped and inverted U-shaped developmental changes in the spontaneous movements of infants via markerless video analysis
Source: Sci Rep. 2020 Oct 8;10:16827. doi: 10.1038/s41598-020-74006-y (PMC7545215; doi:10.1038/s41598-020-74006-y)
Supplement: Supplementary file 1 — Supplementary Information. [file 41598_2020_74006_MOESM1_ESM.pdf]

*Supplementary material*

# Longitudinal Assessment of U-shaped and Inverted U-shaped Developmental Changes in the Spontaneous Movements of Infants via Markerless Video Analysis

Naoki Kinoshita[1], Akira Furui[1,*], Zu Soh[1], Hideaki Hayashi[2], Taro Shibanoki[3], Hiroki Mori[4], Koji Shimatani[5], Yasuko Funabiki[6], and Toshio Tsuji[1,*]

[1] Department of System Cybernetics, Hiroshima University, 1-4-1 Kagamiyama, Higashi-Hiroshima, Hiroshima 739-8527, Japan

[2] Faculty of Information Science and Electrical Engineering, Kyushu University, 744 Motooka, Nishi-Ku, Fukuoka, Fukuoka 819-0395, Japan

[3] College of Engineering, Ibaraki University, 4-12-1 Nakanarusawa, Hitachi, Ibaraki 316-8511, Japan

[4] Future Robotics Organization, Waseda University, 1-104 Totsukamachi, Shinjuku-Ku, Tokyo 169-8050, Japan

[5] Department of Physical Therapy, Prefectural University of Hiroshima, 1-1 Gakuen, Mihara, Hiroshima 723-0053, Japan

[6] Graduate School of Human and Environmental Studies, Kyoto University, Yoshida-Nihonmatsu-Cho, Sakyo-Ku, Kyoto, Kyoto 606-8507, Japan

## The PDF file includes:

**Supplementary Text.** Calculation of movement analysis indices.

**Supplementary Figure S1.** Examples of the motor alteration in Participants C–I.

**Supplementary Figure S2.** Examples of the power of COG fluctuations in Participants C–I.

## Supplementary Text
## Calculation of movement analysis indices

### I. Movement magnitude

Movement magnitude is evaluated using three indices.

${}^{(A_k)}I_1$: Movement frequency
${}^{(A_k)}I_2$: Movement strength
${}^{(A_k)}I_3$: Movement count

Movement frequency ${}^{(A_k)}I_1$ is defined as the ratio of movement time between $1 \le l \le L$ by the following equation:

$$ {}^{(A_k)}I_1 = \frac{1}{L}\sum_{l=1}^{L} {}^{(A_k)}\kappa_l \times 100 \,, $$

$$ {}^{(A_k)}\kappa_l = \begin{cases} 1 & ({}^{(A_k)}M_l \ge M_{\mathrm{th}}) \\ 0 & ({}^{(A_k)}M_l < M_{\mathrm{th}}) \end{cases}, $$

where $M_{\mathrm{th}}$ represents the threshold value for determining the presence or absence of movement. Movement strength ${}^{(A_k)}I_2$ is defined as the strength of movement per unit frame by the following equation:

$$ {}^{(A_k)}I_2 = \frac{1}{L'}\sum_{l=1}^{L} {}^{(A_k)}v_l \,, $$

$$ {}^{(A_k)}v_l = \begin{cases} {}^{(A_k)}M_l & ({}^{(A_k)}M_l \ge M_{\mathrm{th}}) \\ 0 & ({}^{(A_k)}M_l < M_{\mathrm{th}}) \end{cases}, $$

where $L'$ is the sum of the number of frames when ${}^{(A_k)}M_l \ge M_{\mathrm{th}}$. Let ${}^{(A_k)}l^{\mathrm{st}}$ be the frame when ${}^{(A_k)}M_l \ge M_{\mathrm{th}}$ and let ${}^{(A_k)}l^{\mathrm{ed}}$ be the frame when ${}^{(A_k)}M_l \ge M_{\mathrm{th}}$. The interval $[{}^{(A_k)}l^{\mathrm{st}}, {}^{(A_k)}l^{\mathrm{ed}}]$ is defined as one movement; the movement count ${}^{(A_k)}I_3$ is calculated by the following equation:

$$ {}^{(A_k)}I_3 = \frac{Q}{L}, $$

where $Q$ represents the total number of movements between $1 \le l \le L$. Additionally, if ${}^{(A_k)}l^{\mathrm{ed}} \ge L$, then ${}^{(A_k)}l^{\mathrm{ed}} = L$.

### II. Movement balance

Movement balance is evaluated by the following three indices:

${}^{(A_{k_1},A_{k_2})}I_4$: Ratio of indices ${}^{(A_{k_1})}I_1$ and ${}^{(A_{k_2})}I_1$
${}^{(A_{k_1},A_{k_2})}I_5$: Ratio of indices ${}^{(A_{k_1})}I_2$ and ${}^{(A_{k_2})}I_2$
${}^{(A_{k_1},A_{k_2})}I_6$: Symmetry in ${}^{(A_{k_1})}M_l$ and ${}^{(A_{k_2})}M_l$

${}^{(A_{k_1},A_{k_2})}I_4$ and ${}^{(A_{k_1},A_{k_2})}I_5$ are calculated from the ratio of movement frequency ${}^{(A_k)}I_1$ and movement strength ${}^{(A_k)}I_2$ between each movement area using the following equation:

$$ {}^{(A_{k_1},A_{k_2})}I_4 = \frac{{}^{(A_{k_1})}I_1}{{}^{(A_{k_2})}I_1}, $$

$$ {}^{(A_{k_1},A_{k_2})}I_5 = \frac{{}^{(A_{k_1})}I_2}{{}^{(A_{k_2})}I_2}, $$

where $A_{k_1}$ and $A_{k_2}$ $(k_1, k_2 = 1, 2, \cdots, 8)$ represent the respective movement areas, except whole-body

movements, and $k_1 \neq k_2$. However, if ${}^{(A_{k_1})}I_1 = 0$ and ${}^{(A_{k_1})}I_2 = 0$, then ${}^{(A_{k_1},A_{k_2})}I_4 = 0$ and ${}^{(A_{k_1},A_{k_2})}I_5 = 0$. ${}^{(A_{k_1},A_{k_2})}I_6$ is a correlation coefficient between each area calculated using normalized cross-correlation expressed by the following equation for motor alteration ${}^{(A_{k_1})}M_l$ and ${}^{(A_{k_2})}M_l$.

$$ {}^{(A_{k_1},A_{k_2})}I_6 = \frac{\sum_{l=1}^{L}({}^{(A_{k_1})}M_l - {}^{(A_{k_1})}M^{\mathrm{avg}})({}^{(A_{k_2})}M_l - {}^{(A_{k_2})}M^{\mathrm{avg}})}{\sqrt{\sum_{l=1}^{L}({}^{(A_{k_1})}M_l - {}^{(A_{k_1})}M^{\mathrm{avg}})^2}\sqrt{\sum_{l=1}^{L}({}^{(A_{k_2})}M_l - {}^{(A_{k_2})}M^{\mathrm{avg}})^2}}, $$

where ${}^{(A_{k_1})}M^{\mathrm{avg}}$ and ${}^{(A_{k_2})}M^{\mathrm{avg}}$ represent the average values of ${}^{(A_{k_1})}M_l$ and ${}^{(A_{k_2})}M_l$, respectively, between $1 \leq l \leq L$.

### III. Movement rhythm

Movement rhythm is evaluated using six indices.

${}^{(A_k)}I_7$: Mean power frequency of ${}^{(A_k)}M$

${}^{(A_k)}I_8$: Second moment around the mean power frequency ${}^{(A_k)}I_7$

${}^{(A_9)}I_{9_{\mathrm{x}}}$, ${}^{(A_9)}I_{9_y}$: Mean power frequency of $\boldsymbol{G}^{\mathrm{v}}$

${}^{(A_9)}I_{10_{\mathrm{x}}}$, ${}^{(A_9)}I_{11_y}$: Second moment around the mean power frequencies ${}^{(A_9)}I_{9_{\mathrm{x}}}$ and ${}^{(A_9)}I_{9_y}$

${}^{(A_9)}I_{11_{\mathrm{x}}}$, ${}^{(A_9)}I_{11_y}$: Mean power frequency of $\boldsymbol{G}^{\mathrm{d}}$

${}^{(A_9)}I_{12_{\mathrm{x}}}$, ${}^{(A_9)}I_{12_y}$: Second moment around the mean power frequencies ${}^{(A_9)}I_{11_{\mathrm{x}}}$ and ${}^{(A_9)}I_{11_y}$

First, a fast Fourier transform is applied to the motor alteration ${}^{(A_k)}M_l$, COG velocity $(G_{l,x}^{\mathrm{v}}, G_{l,y}^{\mathrm{v}})$, and COG fluctuations $(G_{l,x}^{\mathrm{d}}, G_{l,y}^{\mathrm{d}})$. From the distribution of power spectral density $P(f)$, normalized so that the maximum value becomes 1, $s$ ($s = 1, 2, \cdots, S$; $S = L/L_{\mathrm{f}}$) mean power frequency $F_{\mathrm{MPF}}(s)$ and the reciprocal of second moment around mean power frequency $D_{\mathrm{MPF}}(s)$ are calculated for each frequency analysis frame $L_{\mathrm{f}}$ ($L_{\mathrm{f}} \leq L, L/L_{\mathrm{f}} \in \mathbb{N}$).

$$ F_{\mathrm{MPF}}(s) = \frac{\sum_{f=0}^{f_{\max}} fP(f)}{\sum_{f=0}^{f_{\max}} P(f)}, $$

$$ D_{\mathrm{MPF}}(s) = \sqrt{\frac{\sum_{f=0}^{f_{\max}} P(f)}{\sum_{f=0}^{f_{\max}} P(f)\,(f - F_{\mathrm{MPF}}(s))^2}}, $$

where $f_{\max}$ represents the maximum value of the frequency band used for the analysis. ${}^{(A_k)}I_7$, ${}^{(A_9)}I_9$, ${}^{(A_9)}I_{11}$ and ${}^{(A_9)}I_8$, ${}^{(A_9)}I_{10}$, ${}^{(A_9)}I_{12}$ are the average values of each $F_{\mathrm{MPF}}(s)$ and each $D_{\mathrm{MPF}}(s)$, respectively, between $1 \leq s \leq S$.

### IV. COG movement

COG movement is evaluated by the following two indices:

${}^{(A_9)}I_{13_{\mathrm{x}}}$, ${}^{(A_9)}I_{13_y}$: Variation in the COG velocity

${}^{(A_9)}I_{14_{\mathrm{x}}}$, ${}^{(A_9)}I_{14_y}$: Variation in the COG fluctuation

These indices are defined as the average value between $1 \leq l \leq L$ of the COG velocity $(G_{l,x}^{\mathrm{v}}, G_{l,y}^{\mathrm{v}})$ and the COG fluctuations $(G_{l,x}^{\mathrm{d}}, G_{l,y}^{\mathrm{d}})$.

$$ ({}^{(A_9)}I_{13_{\mathrm{x}}}, {}^{(A_9)}I_{13_{\mathrm{y}}}) = (\frac{1}{L}\sum_{l=1}^{L} G_{l,x}^{\mathrm{v}}, \frac{1}{L}\sum_{l=1}^{L} G_{l,y}^{\mathrm{v}}), $$

$$ ({}^{(A_9)}I_{14_{\mathrm{x}}}, {}^{(A_9)}I_{14_{\mathrm{y}}}) = (\frac{1}{L}\sum_{l=1}^{L} G_{l,x}^{\mathrm{d}}, \frac{1}{L}\sum_{l=1}^{L} G_{l,y}^{\mathrm{d}}). $$

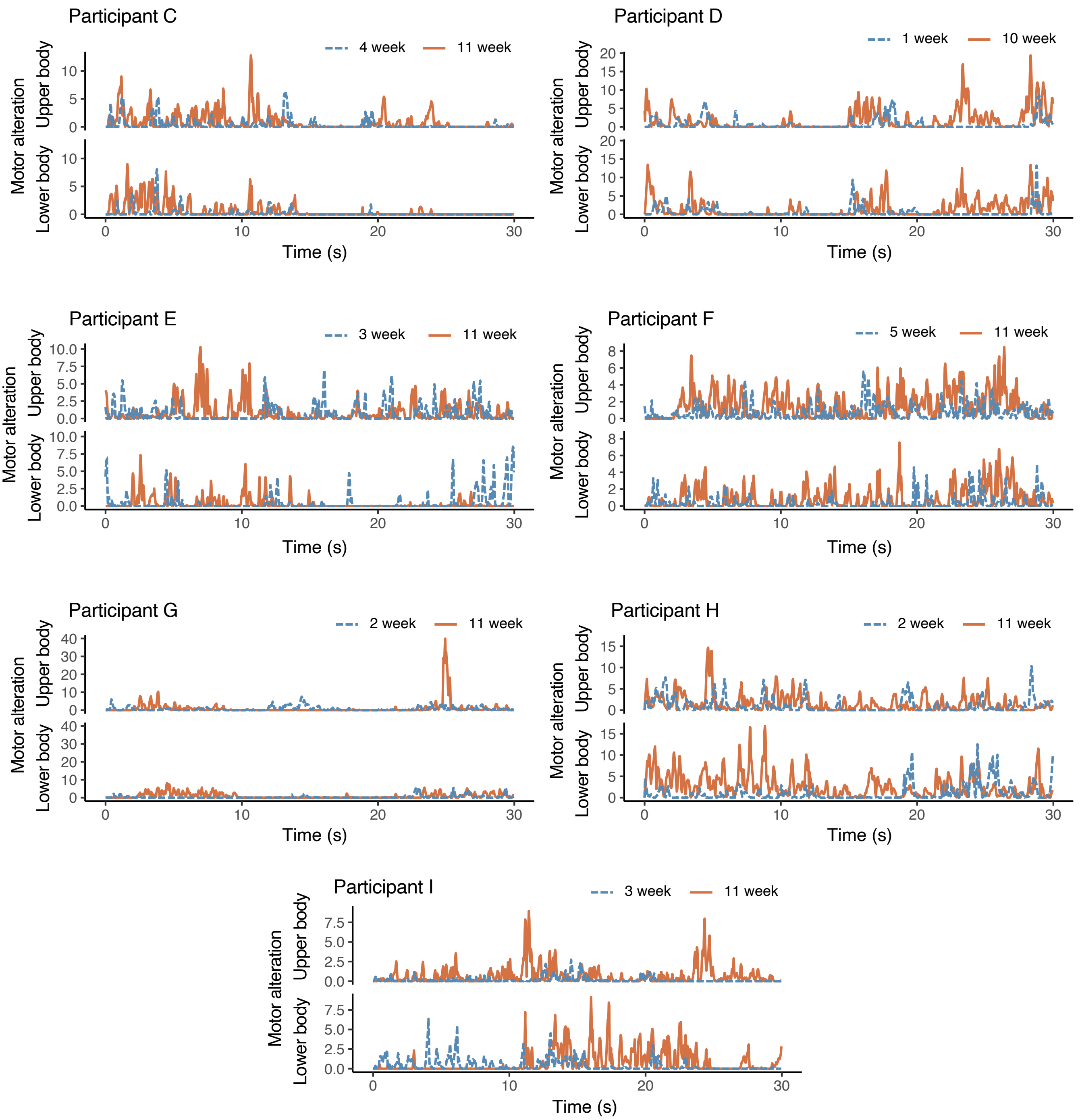


**Supplementary Figure S1.** Examples of the motor alteration in Participants C–I. The waveforms at two time points, i.e., at 1–5 weeks and 10–11 weeks of the corrected ages, are shown. The selection of time points varies according to the number of measurements for each participant.

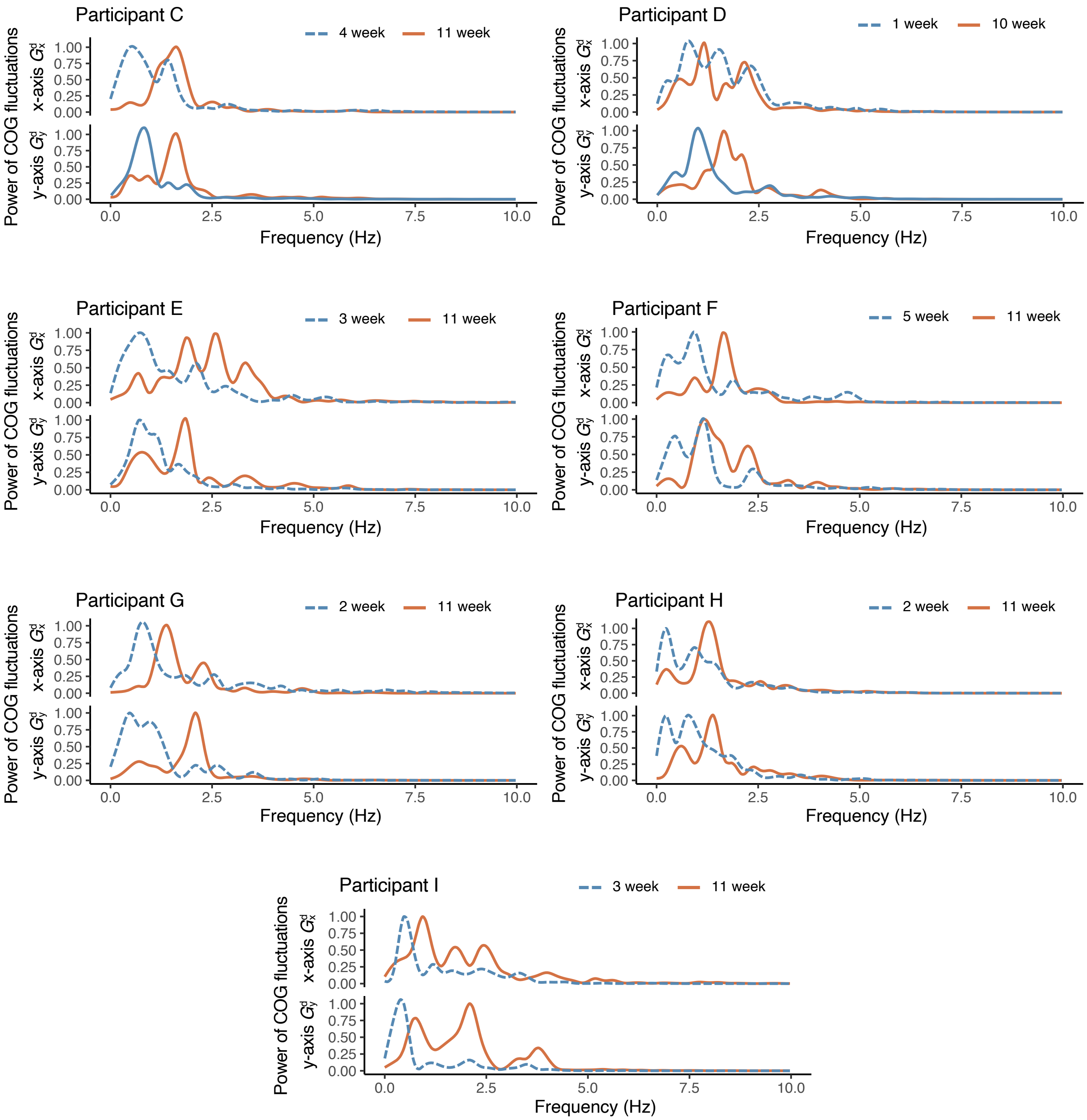


**Supplementary Figure S2.** Examples of the power of COG fluctuations in Participants C–I. Each density is normalized so that the maximum value becomes 1. The waveforms at two time points, i.e., at 1–5 weeks and 10–11 weeks of the corrected ages, are shown. The selection of time points varies according to the number of measurements for each participant.
